# Patients’ Use of the Internet to Find Reliable Medical Information About Minor Ailments: Vignette-Based Experimental Study

**DOI:** 10.2196/12278

**Published:** 2019-11-11

**Authors:** Joyce Kwakernaak, Just A H Eekhof, Margot W M De Waal, Elisabeth A M Barenbrug, Niels H Chavannes

**Affiliations:** 1 Department Public Health & Primary Care Leiden University Medical Centre Leiden Netherlands; 2 Consumentenbond Den Haag Netherlands

**Keywords:** internet, information seeking behaviour, consumer health information, diagnosis, humans, adult

## Abstract

**Background:**

Little is known about the exact process of how patients search for medical information on the internet and what they retrieve. There is especially a paucity of literature on browsing for information on minor ailments, a term used for harmless diseases that are very common in the general population and thus have a significant impact on health care.

**Objective:**

This vignette-based experimental study aimed to explore what kind of Web-based search strategies are applied and how search strategies, demographic characteristics, and the quality of the visited websites relate to finding the right diagnosis. Additional goals were to describe how searching on the Web influences one’s perception of the severity of the potential diagnosis and whether or not the participants would discuss the information they found on the internet with their doctors.

**Methods:**

Out of 1372 survey participants, 355 were randomly sampled, and 155 of them were recruited and assigned to one of four clinical scenarios. Each search term they used was classified as one of three search strategies: (1) hypothesis testing, (2) narrowing within the general hypothesis area, and (3) symptom exploration. The quality of the websites used was determined by using the DISCERN instrument. To compare the diagnostic accuracy of the participants before and after the internet search, a McNemar test was used. Chi-square tests were used to describe which factors are related to the chosen search strategy. A multivariate binary logistic regression model was constructed to predict which factors are related to finding a sound diagnosis after searching the internet for health information.

**Results:**

Most participants (65.8%, 102/155) used the symptom exploration strategy. However, this depends on the assigned scenario (*P*<.001) and the self-estimated severity score of the symptoms before the internet search (*P*=.001). A significant relation was found between choosing an accurate diagnosis and age (odds ratio [OR] 0.94, 95% CI 0.90 to 0.98) and the clinical scenario, as well as the use of high-quality websites (OR 7.49, 95% CI 1.85 to 30.26). Browsing the internet did not lead to a statistically significant change in participants’ beliefs about the severity of the condition (McNemar test, *P*=.85). Most participants (65%) shared their retrieved information with their physician and most of them (75%) received a positive response.

**Conclusions:**

Our findings suggest that most patients use a symptom-based approach; however, if patients expect the potential diagnosis to be severe, they tend to use a hypothesis verification strategy more often and are therefore prone to certain forms of bias. In addition, self-diagnosing accuracy is related to younger age, the symptom scenario, and the use of high-quality websites. We should find ways to guide patients toward search strategies and websites that may more likely lead to accurate decision making.

## Introduction

### Background

Over the last few decades, the internet has become an important and easily accessible source of medical information for patients [1,2]. A reason that people frequently mention for visiting the Web is to find reassurance or to find an explanation or diagnosis for their physical complaints [2]. Furthermore, people tend to use the internet for determining whether or not they should consult a physician [2]. Younger patients, females, and highly educated individuals are known to search for health information more often than others [3,4]. Internet use has the potential to improve patient empowerment, as well-informed patients tend to play a more active role in their own health care [5]. In addition, it may influence the patients’ timing of help seeking behavior [6]. These findings also correspond with the results of a study performed on the widely used website, Thuisarts.nl. This is a reliable source of medical information about minor ailments and advice on self-care, developed by the Dutch College of General Practitioners. Research has shown that in the 2 years after the introduction of this website, the number of short general practitioner (GP) consultations decreased by 12% [7]. However, there are downsides to the use of this easily accessible source of information. It should be considered that most patients are not medically trained. This makes it difficult for them to understand medical jargons and to find accurate information, especially for those with lower education levels and a low socioeconomic status [8,9]. Furthermore, there is an abundance of low-quality websites available, as only a minority meets the standardized quality and accuracy requirements [10,11]. However, no less than two-thirds of the patients have a high level of confidence in the information retrieved from the internet [12]. Using this incorrect information can lead to incorrect decisions [13,14] or to the retrieval of incorrect notions about a medical condition or treatment [15,16]. In addition, lay individuals are often inaccurate when they try to self-diagnose without consulting a physician [17].

### Previous Research

What remains relatively unexplored in the current literature is exactly how patients look for information on the internet. A certain approach was chosen by Pang et al [18], who divided search behaviors into 4 different categories. The distinction was not made to determine the best strategy but to indicate that the following 4 different search strategies have different needs in their search for information: *Quick Fact Seeking* refers to patients terminating their search once they retrieved superficial information for a specific health issue. Therefore, websites should provide key points and a brief summary that is relevant to the topic; *All-Around Skimming* indicates patients who go through a wide range of information in a fast manner. To support this behavior, excerpts and previews will be helpful; *Focused Reading* denotes concentrated reading on a particular topic. Reader-friendly features are recommended to support this behavior; and *Knowledge Digging* indicates the intense reading associated with the in-depth research on a number of diverse health topics. Therefore, a broader range of information should be provided. They created a design for consumer health websites that meets these different needs and concluded that this approach will lead to better knowledge acquisition. Other literature on this topic has been derived from experimental studies, where participants were assigned to search the internet for information on hypothetical scenarios. Keselman et al [19] observed that lay individuals using the *verification of the primary hypothesis* strategy tend to seek out data that correspond to their incorrect initial diagnosis to confirm their own hypothesis and health beliefs (confirmation bias). Participants using the *narrowing search within the general hypothesis* strategy remained indecisive about the diagnosis, whereas the *bottom-up symptom exploration* strategy seemed the most successful. Luger et al [20] conducted a study that revealed that using previous illness experiences and having less existing medical knowledge were associated with choosing an inaccurate diagnosis. Perez et al [21,22] explain that their results correspond with the dual-processing theory, differentiating between system 1 processing (unconscious, initiative, automatic, rapid, low effort) and system 2 processing (conscious, systematic, deliberative, slow, high effort). System 2 processing is associated with higher-quality decision making and is more often applied by individuals with a higher education level and younger age. Another previous study found that patients often select websites of organizations that they consider to be of good reputation or organizations that are domestic, because it makes them feel more confident that they are getting reliable information. On the contrary, participants often avoid websites that have visible advertising or are obviously profit-oriented [23].

### Study Aim

To address the challenging process of obtaining and applying medical information derived from the internet by patients, it is of great importance to understand how these individuals search for information. There is, however, limited information available on this topic. So far, there has been no research into how people search for medical information on the internet about minor ailments, whereas these are common health issues affecting a large part of everyday health care in general practice. The aim of this study was to explore what kind of Web-based search strategies are being used and if they lead to finding a sound diagnosis. Additional objectives are to determine whether the quality of the used websites and certain participant characteristics are related to finding the right diagnosis. Furthermore, this paper describes how searching on the Web influences the perception of severity of the potential diagnosis and if the participants in general would discuss the retrieved information on the internet with their doctor.

## Methods

### Recruitment of Participants

**Figure 1:**
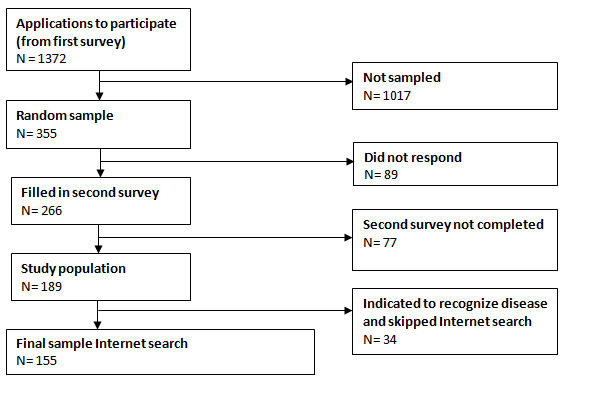
A flowchart of patient recruitment.

This research was conducted on a vignette-based experimental study design that focused on the internet search patterns of Dutch adults. Participants were recruited through a survey on the use of the internet for obtaining information about medical issues, which can be found in Multimedia Appendix 1. This survey was compiled in collaboration with the Dutch Consumers’ Association, which is an independent nonprofit association that conducts research and makes publications about various products, services, and injustice in society. The survey was sent to the Association’s panel members who could fill out the Web-based survey from January 18 to January 27, 2017. This resulted in a total number of 5774 respondents. These participants were asked if they were willing to apply for the experimental test. However, they were only able to do so if they met the inclusion and exclusion criteria. The inclusion criteria were that the participants have access to the internet, that they use it for obtaining information about medical issues, and that they were aged 18 years or older. In addition, the participants were not able to apply if they or their housemates were medically educated. A random sample was taken from the resulting 1372 applicants, which resulted in 355 selected individuals who received an invitation email. In total, 189 of the 355 selected participants were included in this study, of which 155 completed the internet search. The 34 participants who did not complete the internet search indicated at the beginning of the survey that they recognized the disease mentioned in their assigned clinical scenario. Therefore, these participants did not have to do an internet search but were immediately forwarded to the final question in which they had to indicate what they thought was the accurate diagnosis (see Figure 1). The experimental test was run from February 25 until March 12, 2017. The participants did not receive any rewards or payment for their participation but were promised to be informed about the results of the study.

### Study Procedures

Participants were randomly and equally assigned to 1 of 4 clinical scenarios, which represented the symptoms of xanthelasma, seborrheic keratosis, carpal tunnel syndrome (CTS) or benign paroxysmal positional vertigo (BPPV). The scenarios of xanthelasma and seborrheic keratosis consisted of an image, whereas the scenarios of CTS and BPPV were represented through a textual description of the symptoms (see Multimedia Appendix 2). The scenarios were developed with input from clinical coauthors (NHC and JAHE). The reason why these diseases were chosen is because they belong to the so-called *minor ailments*. This term is used for relatively harmless diseases that are incredibly common in the general population and thus have a significant impact on health care. However, some of the symptoms can also occur in other diseases. As a result, they are not immediately recognizable for a medically untrained individual. Participants were not informed that the symptoms were suggestive of xanthelasma, seborrheic keratosis, CTS, or BPPV. The remaining 155 participants had to fill out a second survey at home concerning the assigned scenario and related questions (see Multimedia Appendix 3). After receiving their scenario, the participants had to choose their initial possible diagnosis that would explain the symptoms mentioned in their scenario. They also had to indicate whether they estimated that the disease was severe or not severe. The participants were then instructed to search the internet using the Web browser, Google, as though they were experiencing these symptoms themselves. Their internet search was recorded by means of print screens and search terms, which the participants recorded themselves. After conducting the internet search, they had to choose a final explanatory diagnosis, severity score, and answer questions such as on which websites they found the information to diagnose the symptoms and whether they would discuss the information found with their physician.

### Data Preparation and Coding of Education Level, Internet Search Behaviors, and Diagnostic Accuracy

Education level was classified as 1 of 3 categories: low, intermediate, and high. Participants who received primary school, lower vocational education, preparatory secondary vocational education, or general secondary education were classified as low educated. The second group who received senior secondary vocational education, senior secondary general education, or preuniversity education was classified as intermediate educated. The ones who received higher professional education or academic higher education were considered highly educated. Each search term that was entered was classified as 1 of 3 search strategies: (1) hypothesis testing, (2) narrowing within the general hypothesis area, and (3) symptom exploration [19,21]. Hypothesis testing means entering relevant search terms to verify a diagnostic primary hypothesis (ie, entering *melanoma*). The second strategy describes narrowing the search within a general hypothesis area (ie, entering *skin conditions*). Symptom exploration refers to entering search terms that involve symptoms (ie, entering *brown, hump, crust on skin*). The participants’ assessments of the diagnosis were coded in 2 categories. These categories were as follows: *accurate* if the stated diagnosis matched the diagnosis of the assigned scenario and *not accurate* if the stated diagnosis did not match or if the participants did not know the answer, were unsure, or guessed multiple diagnoses. Of course, participants were not accounted for spelling errors and both medical and vernacular names were labeled as accurate, provided they were referring to the right condition. Examples of accurate diagnoses that were mentioned by participants who were assigned to the seborrheic keratosis vignette were as follows: *Senile wart* or *seborrheic keratosis*. Examples of inaccurate diagnoses were *birthmark*, *melanoma*, or *don’t know*. To check whether coding was done unequivocally, almost half of the participants were also coded by a second independent team member. Team members met regularly to compare their own independent coding of participants’ search terms and assessments of the diagnosis. The initial assessment of the search terms and diagnosis showed an average difference of 8.1% among team members, but this was resolved completely through discussion until members of the team reached a consensus. The quality of the websites that were used by the participants to find their diagnosis was determined by using the DISCERN instrument [24,25]. Websites were considered to be of low quality if <2, intermediate if between 2.1 and 3.9, and high if >4. Participants could submit up to four websites but were assigned to the group *low*, *intermediate*, or *high* based on the website used with the highest quality. To ensure reliable coding, a selection of 20 websites was independently classified by a second team member. All websites were independently placed in the same group, so no difference in assessment was found.

### Statistical Analysis

The demographic characteristics of the participants were identified with descriptive statistics. A comparison between participants of the 4 different scenarios was made with a one-way analysis of variance (ANOVA) test (for age in years) and chi-square tests (for gender, education level, and self-estimated severity score). A McNemar test was used to compare the diagnostic accuracy of the study population before and after the internet search. Furthermore, a multivariate binary logistic regression model was constructed to predict the choice of an accurate diagnosis after searching on the Web for health information, using search strategy, age, gender, education level, clinical scenario, and the quality of the websites used as predictors. In this analysis, finding an accurate diagnosis served as the dependent variable, with finding the inaccurate diagnosis as the reference group. Finally, a McNemar test was used to compare the self-chosen severity score before and after the internet search. A *P* value lower than .05 was considered significant. All statistical analyses were performed using SPSS version 20 (IBM).

### Ethical Approval

The research plan has been submitted to the Medical Ethical Committee (MEC) of the Leiden University Medical Centre. As the data cannot be traced back to the individual participant, the MEC considered the study exempt.

## Results

### Participant Characteristics

The participant demographic and personal characteristics are presented in Table 1. Participant demographic characteristics were identified with descriptive statistics, and a comparison between participants of the 4 different scenarios was made with a one-way ANOVA test (for age in years) and chi-square tests (for gender, education level, and self-estimated severity score). The study population ranged from 18 to 74 years of age, the overall mean age being 47.5 (SD 13.5) years. There were as many men as women (48% versus 52%, respectively). Most participants were highly educated, considering 65% graduated on a high education level and 28% obtained an intermediate education level. Comparing the 4 different scenarios with each other, the mean age was similar between the 4 groups (*P*=.45). The gender distribution is not exactly equally divided for each scenario. Especially, the scenarios of seborrheic keratosis (70% male), CTS (30% male), and BPPV (36% male) were unequally distributed (*P*=.001). Education level showed a similar distribution pattern for all 4 scenarios (*P*=.52). There was a difference in the self-estimated severity score before the internet search between the 4 different scenarios; particularly, the participants who were assigned to the seborrheic keratosis scenario tend to score the symptoms as *severe* (61%, *P*<.001).

**Table 1:** Participant demographic characteristics (N=155).

Characteristics per scenario		Xanthelasma (N=38)	Seborrheic keratosis (N=44)	CTS^a^ (N=37)	BPPV^b^ (N=36)	Total (N=155)	*P* value
Age (years), mean (SD)		50.4 (12.5)	47.1 (14.5)	47.2 (14.0)	45.3 (12.7)	47.5 (13.5)	.45
**Gender, n (%)**							**.001**
	Male	19 (50)	31 (70)	11 (30)	13 (36)	74 (48)	
	Female	19 (50)	13 (30)	26 (70)	23 (64)	81 (52)	
**Education level, n (%)**							**.52**
	Low	2(5)	6 (14)	1 (3)	2 (6)	11 (7)	
	Intermediate	12 (32)	12 (27)	11 (30)	8 (22)	43 (28)	
	High	24 (63)	26 (59)	25 (68)	26 (72)	101 (65)	
**Self-estimated severity score, n (%)**							**<.001**
	Severe	2 (5)	27 (61)	11 (30)	12 (33)	52 (34)	
	Not severe	36 (95)	17 (39)	26 (70)	24 (67)	103 (66)	

^a^CTS: carpal tunnel syndrome.

^b^BPPV: benign paroxysmal positional vertigo.

### Effect of Internet Searching on Diagnostic Accuracy

**Figure 2:**
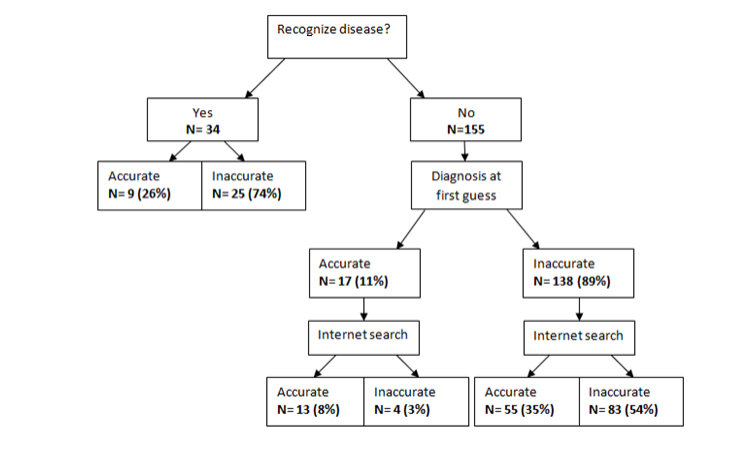
The effect of internet use on self-diagnosing accuracy (N=189).

Of the 189 participants, 34 indicated that they recognized the condition that caused the symptoms of the scenario, and they provided a final diagnosis without doing an internet search; only 9 out of 34 (26%) were accurate (see Figure 2). The remaining 155 participants also had to provide a first diagnosis before searching for information on the internet, of which 17 (10.9%) were accurate. After the internet search, 4 participants (4/155, 2.5%) found an incorrect diagnosis, even though their initial diagnosis was correct. Of the 138 participants who were initially inaccurate, 35.4% (55/155) found the right diagnosis. A McNemar test revealed that performing an internet search leads to a statistically significant improvement in self-diagnosing accuracy compared with before the internet search (44% versus 11%, respectively; *P*<.001).

### Search Strategies Used

Overall, most of the participants used the symptom exploration strategy to find a diagnosis (66%). Hypothesis testing was the second most frequently used strategy (23%), whereas the least used strategy was narrowing in the general area (12%). The used search strategy depends on the assigned scenario. A chi-square test confirms that there is a statistically significant correlation between the assigned scenario and the chosen search strategy (*P*<.001). The scenarios xanthelasma, CTS, and BPPV were predominantly solved using the symptom exploration strategy (68%, 92%, and 92%, respectively). Hypothesis testing was the most used strategy among the participants who were assigned to the seborrheic keratosis scenario (61%). In addition, there was a difference in the self-estimated severity score, confirmed by a chi-square test that reveals that the chosen search strategy is significantly related to the self-estimated severity score of the symptoms before the internet search (*P*=.001). Of the participants who used the hypothesis testing strategy, 60% scored the symptoms as *severe*, in advance. The participants who used the narrowing strategy and symptom exploration strategy only scored the symptoms as *severe* in 22% and 26% of the cases, respectively.

### Characteristics That Influence Diagnostic Accuracy

The mean age of the participants who were accurate in their final diagnosis was 45.3 (SD 13.0) years. Of the participants who were inaccurate, this was 49.2 (SD 13.7; see Multimedia Appendix 4). Of the females, 52% chose the correct diagnosis, compared with 35% of the men. Furthermore, especially those participants who were assigned to diagnose xanthelasma and CTS were accurate in their diagnosis (66% and 68%, respectively). Seborrheic keratosis was the most difficult to diagnose, as only 20% chose an accurate diagnosis. In addition, users of the hypothesis testing strategy were accurate in 31% of the cases, whereas narrowing in the general area strategy led to 33% correct answers. The symptom exploration strategy led to an accurate diagnosis more often, as 50% of the individuals who used this strategy found the accurate diagnosis. Of the 196 visited websites, 6 could not be traced back by the assessor (JK). Therefore, 190 websites were checked on quality using the DISCERN instrument. Almost all visited websites were written in Dutch (95%), the remaining 5% were written in English. Of the participants who used only low-quality websites or intermediate-quality websites, 52% and 34%, respectively, found the accurate diagnosis. The participants who used at least one high-quality website were more accurate, for 64% gave a correct diagnosis. A multivariate binary logistic regression model was constructed to predict the diagnostic accuracy that was based on information retrieved from the internet. No multicollinearity was found. The resulting model showed a significant association between diagnostic accuracy with age, clinical scenario, and the quality of the websites used (see Table 2). For every 1-year increase in age, the odds of choosing the accurate diagnosis decreased by 6% (odds ratio [OR] 0.94, 95% CI 0.90 to 0.98). Furthermore, the clinical scenarios xanthelasma and CTS were significantly associated with choosing the right diagnosis (OR 10.73, 95% CI 3.24 to 35.54 versus OR 2.74, 95% CI 1.08 to 6.96, respectively) in contrast to the scenarios seborrheic keratosis (OR 0.22, 95% CI 0.07 to 0.71) and BPPV (OR 0.15, 95% CI 0.06 to 0.41), which are significantly associated with choosing an inaccurate diagnosis. Another factor significantly related to finding the right diagnosis is the highest quality of the websites used on which the participant found the final diagnosis. Compared with participants who only used low-quality websites, participants who used at least one high-quality website were most likely to find the accurate diagnosis (OR 7.49, 95% CI 1.85 to 30.26), followed by intermediate-quality website users (OR 1.53, 95% CI 0.33 to 7.00). In addition, there was no significant association with gender, education level, and search strategy.

**Table 2:** Multivariate binary logistic regression analysis of the relationship of demographic characteristics with reliance on choosing the accurate diagnosis after an internet search (N=155).

Characteristics		Odds ratio (95% CI)	*P* value
Age (years)		0.94 (0.90-0.98)	.005
**Gender**			**.12**
	Male	1 (reference)	
	Female	2.36 (0.80-6.97)	
**Education level**			**.82**
	Low	1 (reference)	
	Intermediate	0.81 (0.09-7.21)	
	High	0.62 (0.09-4.31)	
**Scenario^a ^**			**<.001**
	Xanthelasma	10.73 (3.24-35.54)	
	Seborrheic keratosis	0.22(0.070-0.71)	
	CTS^b^	2.74 (1.08-6.96)	
	BPPV^c^	0.15 (0.06-0.41)	
**Search strategy^a ^**			**.82**
	Hypothesis testing	1.41 (0.48-4.18)	
	Narrowing	0.83 (0.24-2.81)	
	Symptom exploration	0.86 (0.33-2.21)	
**Quality of websites used**			**.005**
	Low	1 (reference)	
	Intermediate	1.53 (0.33-7.00)	
	High	7.49 (1.85-30.26)	

^a^The odds ratios of *Scenario* and *Search strategy* were derived by settings, CONTRAST subcommand deviation. The effect for each category of the independent variable is compared with the overall mean.

^b^CTS: carpal tunnel syndrome.

^c^BPPV: benign paroxysmal positional vertigo.

### Influence of Searching the Internet on Self-Estimated Severity Score

Before searching the internet, 34% of the participants tend to score the symptoms of the assigned scenario as *severe*. After finding information about the possible diagnosis on the internet, 34% still scored the symptoms as *severe*. Therefore, searching the internet did not lead to a statistically significant change in participants’ beliefs about the severity of the condition (McNemar test, *P*=.85). This last self-chosen severity score after the internet search was related to the assigned scenario (chi-square test, *P*=.002) but was not related to the diagnostic accuracy (chi-square test, *P*=.13).

### Whether or Not to Discuss With the Physician?

In general, almost two-thirds (65%) of the participants have discussed medical information found on the Web with their physician in the past. In 75% of these consultations, the participants received a positive response from the physician. The most frequently heard comments from the physicians to their patients were that searching the internet contributes to well-informed patients and that it makes them better prepared for the consultation. Furthermore, it was mentioned that the physicians need to explain less and therefore the consultation would take less time. Only 5% received a negative response, which mainly consisted of warnings that the information on the internet is not always correct and that self-diagnosing by laymen is undesirable. Of the other participants who did not share the information found on the Web with their physician, most did not have a special reason for not telling or had simply forgotten to do so. Only 3 participants mentioned that they expected that, after sharing, the physician would no longer look at them with an *open mind*.

## Discussion

### Principal Findings and Comparison With Prior Work

With our vignette-based experimental study, we tried to provide an initial insight into how patients search for medical information on the internet and how they attempt to self-diagnose symptoms of minor ailments on the Web. Despite the fact that Web-based self-diagnosis is very popular, limited research has been done on this topic, especially regarding internet search on minor ailments, even though these ailments are common health issues and therefore a very common reason for consulting the GP. Our findings suggest that searching on the Web can be a helpful tool in the process of self-diagnosing. Overall, 44% of the participants were accurate in their final diagnosis, which was a significant improvement compared with the 11% who were accurate before searching the internet. On the contrary, it should also be considered that 3% of the participants who were initially accurate found an incorrect diagnosis afterward. Of the participants who stated they recognize the diagnosis in advance without the help of the internet, the self-diagnosing accuracy was poor and only 26% were accurate.

To determine why some participants find the right diagnosis whereas others do not, we observed how people search for medical information on the internet and which factors or characteristics contribute to finding the accurate diagnosis. In the overall study group, most people tend to use a symptom exploration strategy. Hypothesis verification strategy and narrowing in the general area strategy were used less. This corresponds with previous research on internet search behaviors of emergency department patients and a study on internet queries, in which it is also shown that most of the internet searches are focused on symptoms [17,26]. Which strategy was chosen seems to be associated with the type of scenario and how severe the participants estimated that the diagnosis would be in advance. For example, a lot of participants assigned to the seborrheic keratosis scenario filled in an initial diagnosis of melanoma or skin cancer and estimated that the diagnosis would be severe. A significant majority of the *keratosis* participants chose the hypothesis testing strategy. This is in line with other previous research that showed that more focused seekers usually have a clear idea and a plan to research in a limited set of results (hypothesis testing), whereas more exploratory seekers usually try to address unfamiliar problems by retrieving a wider range of information (symptom exploration). Patients who are searching for information about medical issues tend to use a more exploratory method, especially individuals who need to handle health issues of themselves or their loved ones and individuals who have a high level of uncertainty about the subject [27]. After the internet search for the seborrheic keratosis scenario with the hypothesis testing strategy, many participants still thought the diagnosis would be skin cancer, and this scenario had the most inaccurate participants compared with the other three. This phenomenon was observed more often in previous research and is known as confirmation bias (starting with a hypothesis and only looking at information that confirms the initial hypothesis) and premature termination bias (quitting the search after viewing only 1 topic) [19,28]. These studies show that especially hypothesis-driven strategies are prone to these forms of bias.

Furthermore, the multivariate binary logistic regression analysis showed that younger age, the symptom scenario xanthelasma and CTS, and the use of higher-quality websites lead to more self-diagnosing accuracy. In the current literature, younger age and higher education are characteristics that contribute to search strategies that are more successful in finding an accurate diagnosis [21]. Our study confirms that a younger age is significantly related to diagnostic accuracy. A possible explanation for this is that younger people have more experience with browsing the internet and that this contributes to finding the right diagnosis [20]. However, the literature is contradictory, as another study stated that more internet experience was not significantly related to diagnostic accuracy [19]. Our study did not find a significant relation with higher education but that may be caused by the fact that few lower educated participants were included in our study population (see Limitations section). It could be based on coincidence that our lower educated individuals just did well self-diagnosing, and therefore we could not detect a significant difference compared with the higher educated participants. A possible explanation for why the scenarios xanthelasma and CTS were easier for the participants to diagnose is because the differential diagnosis of these symptoms is less extensive and therefore possibly easier to find on the internet. Seborrheic keratosis was the most difficult to diagnose, as only 20% chose an accurate diagnosis. Despite the unequal distribution in gender in this scenario, no difference was found between men (19% accurate) and women (23% accurate). Furthermore, we found that participants who used higher-quality websites were more likely to choose the accurate diagnosis. After studying the list of websites used, it can be concluded that the websites that are considered to be of high quality according to the DISCERN method have often been developed by hospitals or doctors’ associations or departments. Examples are thuisarts.nl (Dutch College of General Practitioners) or oogartsen.nl (Dutch foundation for ophthalmologists of top clinical hospitals). The websites classified as low quality are mainly health forums, where everyone can write anything and there is little or no control over the quality of this information, for example, Artikelsite.info or Mens-en-gezondheid.infonu.nl. This demonstrates that it is of great importance to guide lay people in their internet search by offering websites with reliable information that has been written or verified by professionals. This concept corresponds to previous research that examined the possible barriers and needs of patients searching for medical information on the internet [29]. One important finding was that patients have indicated to prefer guidance of health professionals to find appropriate Web-based resources [29,30]. Furthermore, the findings suggest that greater involvement by health professionals could contribute to an improved relationship between the health professional and patient by minimizing barriers such as finding incomprehensible information, information in medical jargon, volume of information, and inconsistency of information across different sources [29]. We suggest that health professionals can play a role in consumers’ navigation of Web-based health information by, for example, placing weblinks to high-quality websites on the internet homepage of the doctor’s practice or on the information board that is often present in the waiting room of the practice.

What falls outside the scope of our study is what role search engines play in managing the displayed content and what impact this has on the decision-making process of the consumer. People often only view the top search results and what is shown there is logically very important for information gathering. Some recent researches show that the bias of search engines or social media can have an effect on people [31,32]. There is evidence that obscuring the true identity of an information source, obscuring the affiliations of an information source, and control over user-generated content can greatly influence consumer health knowledge and behavior [31]. It is logical to think that this can also influence the self-diagnosing process, but how exactly is an important subject for future research.

In addition, a frequently mentioned reason why people search for Web-based medical information is to find reassurance [2]. However, the results of the survey show that the self-estimated severity score in advance does not differ from the self-estimated severity score after searching the internet, and this is not related to whether the participants find the right diagnosis or not. Therefore, our study shows that people actually do not find the reassurance they are looking for, even if they find the right diagnosis. Apparently, a visit to the doctor is still necessary. Almost two-thirds of the participants indicated that they would share the information they found with their physician. Previous research supports these findings and indicates that the most frequently mentioned reason to discuss the information found with their physician is to ascertain the opinions of health professionals on the retrieved health information [30]. Most participants received a positive response from their doctor.

### Limitations

There are factors that limit the generalization of the findings. First, the surveys were conducted among panel members of the Dutch Consumers’ Association. This is an independent nonprofit association that conducts research and makes publications about various products, services, and injustice in society. Anyone can become a member of this organization; however, one can expect that these individuals are more conscious and are higher educated compared with the rest of the Dutch population. By selecting these subjects, in particular, a form of selection bias occurs. That would explain why our study population is so highly educated. As some studies show that finding the right diagnosis is related to a higher level of education, it is expected that finding the right diagnosis in the general population is even more difficult than finding the right diagnosis in our research group. Second, participants were instructed to do a scenario-based internet search and were therefore driven by symptoms they did not experience at that moment. Furthermore, in reality it is possible that the course of a disease is gradual and has different stages (eg, CTS). Therefore, patients may experience different symptoms over time instead of perceiving all the symptoms at the same time. This can make the search for the correct diagnosis more difficult in real life. These 2 factors might influence the generalizability of our results, as it may have artificially influenced participants’ search efforts and strategies. Finally, it should be considered that the chosen search strategy and whether patients find the right diagnosis or not depends on the disease and experienced symptoms. Therefore, we realize that only these 4 chosen scenarios cannot represent how patients search the internet for medical information in general, but it does provide an indication.

### Conclusions

Our findings suggest that most patients who search the internet for medical information use a symptom-based approach, but this depends on the experienced symptoms. If the patient expects the potential diagnosis to be severe, they tend to use a hypothesis verification strategy more often and are therefore prone to certain forms of bias. To prevent this, doctors should advise their patients to look for symptoms rather than hypothesis-driven strategies. In addition, self-diagnosing accuracy is related to younger age, the symptom scenario, and the use of higher-quality websites. Although it is difficult to tackle the abundance of low-quality websites, doctors should focus on ways to guide patients toward professional websites that may be more likely to lead to accurate decision making. This can be archived, for example, by placing weblinks to high-quality websites on the internet homepage of the doctor’s practice or on the information board that is often present in the waiting room of the practice. However, future research that includes more patients and more different types of scenarios will be necessary to further understand the complex coordination between patient search strategies, finding reliable websites, and Web-based symptom information processing.

## Appendix

Multimedia Appendix 1 First survey Dutch Consumers’ Association.

Multimedia Appendix 2 Clinical scenarios.

Multimedia Appendix 3 Second survey internet search.

Multimedia Appendix 4 Table with characteristics by accuracy of diagnosis.

## Abbreviations

ANOVA: analysis of variance
BPPV: benign paroxysmal positional vertigo
CTS: carpal tunnel syndrome
GP: general practitioner
MEC: Medical Ethical Committee
OR: odds ratio
