# Supplementary material for: Patients’ Use of the Internet to Find Reliable Medical Information About Minor Ailments: Vignette-Based Experimental Study
Source: J Med Internet Res. 2019 Nov 11;21(11):e12278. doi: 10.2196/12278 (PMC6878104; doi:10.2196/12278)
Supplement: Multimedia Appendix 1 [file jmir_v21i11e12278_app1.pdf]

Appendix 1. First survey Dutch Consumers' Association

1. Do you ever search for medical information on the Internet if you experience health problems yourself?
a. Yes -> go to question 3
b. No
c. Not applicable -> end of questionnaire *
* Thank you for your cooperation. We have no further questions. You can close the window.

2. Why would you decide not to search for medical information on the Internet in case of health problems?
Multiple answers possible. <Answers randomly offered>
a. Make little use of the Internet
b. My computer skills fail
c. It is difficult to determine which information is reliable
c. There is too much medical information on the Internet
e. Preferably go to the (family) doctor
f. Am afraid that I conclude from the information that I have a serious illness
g. Am afraid that the information I found, makes me falsely feel reassured.
h. Otherwise, namely….
i. No special reason
j. I do not know / no opinion
<all to question 16>

3. How do you determine whether or not the found medical information is reliable?
Multiple answers possible.
a. If I see the same information on multiple websites, I assume that it is correct
b. I check if there is a reliable organization behind the website
c. I look at websites that I think are reliable
d. I look at website (s) that my physician has recommended
d. Otherwise, namely…
e. I do not know
<if 3 = c -> submit question 4>

4. Which websites with medical information do you find reliable?
You can enter a maximum of 3 websites. <Not required>
a.………………..
b. ....
c. ....

5. Did you search for medical information on the Internet during the past 12 months because of <u>your own</u> health problems?
a. Yes
b. No -> end of questionnaire *
* Thank you for your cooperation. We have no further questions. You can close the window.

6. Why were you looking for medical information on the Internet?
Multiple answers possible. <Answers randomly offered >
a. Wanted to know what I could do about certain complaints myself
b. Found it difficult to go to the physician with some complaints/questions
c. Was willing to prepare myself before I (possibly) went to the physician
d. The physician gave me too little information
e. I forgot to ask certain questions at the physician

f. Wanted to learn more about other people's experiences in the same medical situation
g. Wanted to avoid costs of possible medication and/or follow-up research
h. Wanted to know if it was necessary to go to the physician
i. Wanted to know more about certain treatments
j. Wanted to know more about the use of medicines (for example side effects)
k. Had little time to go to the physician
l. To reassure myself
m. On the advice of the physician
n. Otherwise, namely …….
o. No special reason
p. I do not know / no opinion

The following questions are about the last time you searched medical information for yourself on the Internet.

7. Which of the following steps did you take based on the medical information you found on the Internet? Multiple answers possible.
a. I went to see a General Practitioner (GP)
b. I went to see a medical specialist
c. Decided not to go to the physician, but to treat / resolve it myself
d. Asked the physician to prescribe a specific medication
e. Asked the physician to perform a specific additional (laboratory) test
f. Decided not to follow the advice of the physician
g. Decided to stop using a certain medication / follow a treatment
h. Adjusted my lifestyle (for example more exercise / healthier eating)
i. Used self-care products such as painkillers, cough liquor, vitamins
j. Other, namely ...
k. No follow-up steps taken
<Note: answers a and c cannot work together>

<a: to question 10
b: to question 14
c: to question 8 (if only answer or in combination with g, h, i)
d: to question 14
e: to question 14
f: to question 14
g: to question 8 (in combination with c but also with h and i) (not if one of the answers a, b, d, e, f, j is given and not as the only answer, then to question 14)
h: to question 8 (if only answer or in combination with c, g, i) (not if one of the answers a, b, d, e, f, j is also given)
i: to question 8 (if only answer or in combination with c, g, h) (not if one of the answers a, b, d, e, f, j is also given)
j: to question 14
k: to question 8>

8. You did not go to see the GP based on the found information. Did you regret it later?
a. Yes
b. No

9. Space for any explanation
.... <large input field, not required>
<all to question 14>

10. Did you tell the GP that you searched the Internet for medical information?
a. Yes
b. No -> to question 12
c. I do not know (anymore) / do not want to mention -> to question 13

11. What was his / her reaction?
a. Positive, namely .... <not required>
b. Negative, namely .... <not required>
c. I do not know (anymore) / I do not want to mention
<all to question 13>

12. Why did you not tell the GP that you searched the Internet for medical information?
Multiple answers possible.
a. Did not want to give the GP the idea that I did not trust him / her
b. I expected that the GP would no longer look at me with an 'open mind'
c. I expected that he / she did not like that
d. I knew he/she did not like that
e. Otherwise, namely………….
f. No special reason

13. Did the GP's findings correspond with the information you found on the Internet?
a. Yes, entirely
b. Yes, for the most part
c. No
d. I do not know (anymore) / I do not want to mention

14. Are you yourself or is a roommate working in the healthcare sector?
a. Yes
b. No -> to question 16a

15. Which specific profession in health care is that?
.... <entry field, not required>

16A. Do you have any comments on this subject or about this questionnaire?
a. Yes, namely ....
b. No

B. You are a:
a. Man
b. Woman

C. What is your age?
.... year

D. What is the composition of your household?
a. Single
b. Cohabiting/ married couple without child(ren) living at home
c. Cohabiting/ married couple with child(ren) living at home
d. Single parent household with child(ren) living at home
e. Different

E. What is your highest education level?
a. Primary education

b. LBO / VMBO high school
c. MAVO high school
d. MBO
e. HAVO/ VWO high school
f. HBO
g. WO / university
h. Different

F. To make an accurate geographical layout, we would like to know your full zip code:
a. Zip code: ... ...
b. I would rather not mention it

You answered all the questions. Click on 'Send' to send your answers.
Thank you for your cooperation.
