# Supplementary material for: Patients’ Use of the Internet to Find Reliable Medical Information About Minor Ailments: Vignette-Based Experimental Study
Source: J Med Internet Res. 2019 Nov 11;21(11):e12278. doi: 10.2196/12278 (PMC6878104; doi:10.2196/12278)
Supplement: Multimedia Appendix 2 [file jmir_v21i11e12278_app2.pdf]

Appendix 2. Clinical scenarios

Xanthelasma

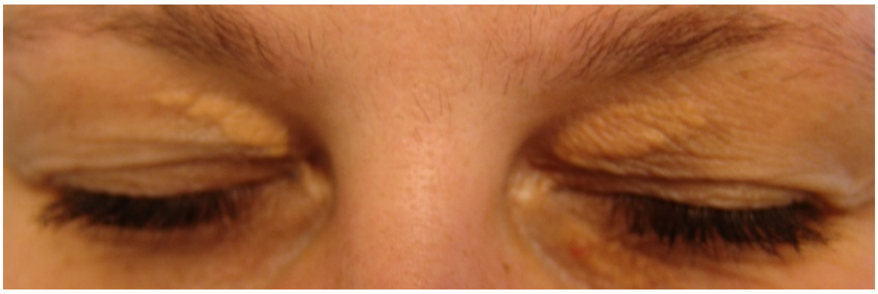

Seborrheic keratosis

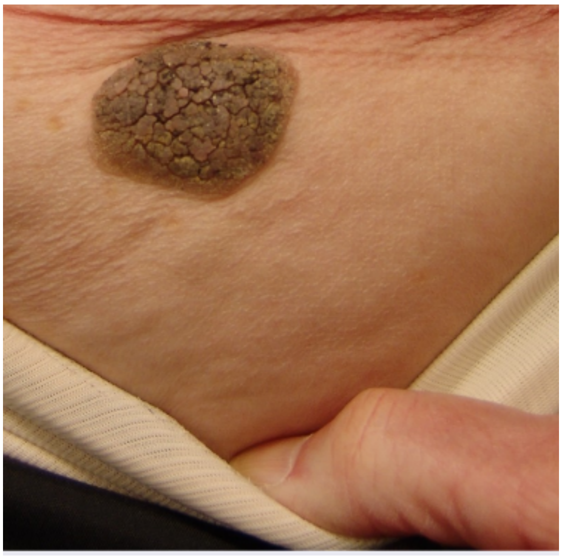

Carpal tunnel syndrome (translated from Dutch)

You are occasionally wake up at night by a tingling feeling in your right hand. Sometimes your hand also hurts a bit. Sometimes you also have these sensations during the day. When performing certain tasks, you notice that the strength and sensitivity of your hand is a little diminished. For example, you have trouble with writing or wringing a dishcloth. If you pay close attention, you notice that you mainly experience these symptoms in your thumb, index finger, and middle finger.

Benign paroxysmal positional vertigo (translated from Dutch)

One morning when you wake up and try to get out of bed, you are suddenly struck by severe dizziness. It makes you feel sick. After lying down for a while you notice that you are feeling better, but when you try to get up again the dizziness immediately returns. You sit down for a while and you feel the dizziness ebb away. Because you feel better again, you decide to get up. During the course of day, you notice the dizziness shows up again with every unexpected turn of the head. The following night you notice that you also feel dizzy when you are lying down, especially when you turn around in bed.
