# Supplementary material for: Patients’ Use of the Internet to Find Reliable Medical Information About Minor Ailments: Vignette-Based Experimental Study
Source: J Med Internet Res. 2019 Nov 11;21(11):e12278. doi: 10.2196/12278 (PMC6878104; doi:10.2196/12278)
Supplement: Multimedia Appendix 3 [file jmir_v21i11e12278_app3.pdf]

Appendix 3. Second survey Internet search

We first ask you to answer two general questions about the following condition. You are NOT allowed to search on the Internet.

Show <image condition> OR <description of the condition>

1. In the first instance, what do you think is the right diagnosis of this condition?
a. <entry field, not required>
b. I do not know

2. Do you recognize this condition?
a. Yes, because .... -> to question 9
b. No

3. To what extent do you think that there is a serious situation?
a. Very serious
b. Reasonably serious
c. Not so serious
d. Innocent
e. I do not know

<no longer able to return to the previous question>
Show <image condition> OR <description of the condition>

Start Google.nl now and enter a search term.

4. Which search term do you start with?
<entry field>

5. After typing in your first search term, create an image/print screen of the entire screen with the search results. Save this document. Make sure you have as many search results as possible on the screen. If you use a tablet, keep the screen vertical.
You can upload the document with the search results here ......

6. Do you think you have found the right diagnosis with your search term?
a. Yes -> to question 9
b. No

7. Try to find the diagnosis with a different search term. Which search term do you use now?
<entry field>

8. After typing in your second search term, create an image/print screen of the entire screen with the search results. Save this document. Make sure you have as many search results as possible on the screen. If you run the job on a tablet, keep the screen vertical.
You can upload the second document with the search results here ......

9. What do you think is the right diagnosis of this condition?
a. <large input field>
b. I do not know ->end questionnaire *)

*) Thank you for your cooperation. We have no further questions. You can close this window.

10. On which website (s) did you find your diagnosis?
a. .... <not required>
b. .... <Not required>
c. .... <Not required>
d. .... <Not required>

11. To what extent do you consider your (found) diagnosis to be serious?
a. Very serious
b. Reasonably serious
c. Not so serious
d. Innocent
e. I do not know

12. Suppose you would have the condition yourself. What would you do based on the information you found?
a. Make an appointment with the GP in the short term
b. Discuss it with the doctor once
c. Wait
d. Use self-care product, namely ....
e. Otherwise, namely ……………..
f. I do not know

You answered all the questions. Click on 'Send' to send your answers.
Thank you for your cooperation.
