# Supplementary material for: Patients’ Use of the Internet to Find Reliable Medical Information About Minor Ailments: Vignette-Based Experimental Study
Source: J Med Internet Res. 2019 Nov 11;21(11):e12278. doi: 10.2196/12278 (PMC6878104; doi:10.2196/12278)
Supplement: Multimedia Appendix 4 [file jmir_v21i11e12278_app4.pdf]

Appendix 4. Table with characteristics by accuracy of diagnosis

Characteristics	Accurate diagnosis n(%)/ mean(SD) N= 68	Inaccurate diagnosis n(%)/mean(SD) N=87	Total n(%)/mean(SD) N=155
**Age**	45,3 (13,0)	49,2 (13,7)	47.5 (13.5)
**Gender**			
- **Male**	26 (35%)	48 (65%)	74 (100%)
- **Female**	42 (52%)	39 (48%)	81 (100%)
**Education**			
- **Low**	3 (27%)	8 (73%)	11 (100%)
- **Intermediate**	23 (53%)	20 (47%)	43 (100%)
- **High**	42 (42%)	59 (58%)	101 (100%)
**Scenario**			
- **Xanthelasma**	25 (66%)	13 (34%)	38 (100%)
- **Seborrheic keratosis**	9 (20%)	35 (80%)	44 (100%)
- **CTS**	25 (68%)	12 (32%)	37 (100%)
- **BPPV**	9 (25%)	27 (75%)	36 (100%)
**Search strategy**			
- **Hypothesis testing**	11 (31%)	24 (69%)	35 (100%)
- **Narrowing**	6 (33%)	12 (67%)	18 (100%)
- **Symptom exploration**	51 (50%)	51 (50%)	102 (100%)
